# Supplementary material for: Causal Evidence for the Dependence of the Magnitude Effect on Dorsolateral Prefrontal Cortex
Source: Sci Rep. 2018 Nov 8;8:16545. doi: 10.1038/s41598-018-34900-y (PMC6224465; doi:10.1038/s41598-018-34900-y)
Supplement: Supplementary file 1 — Supplementary Information [file 41598_2018_34900_MOESM1_ESM.docx]

**Supplementary Information**

**Causal Evidence for the Dependence of the Magnitude Effect on Dorsolateral Prefrontal Cortex**

**Ian C. Ballard^1,2,3^, Gökhan Aydogan^4^, Bokyung Kim^3^, Samuel M. McClure^4^**

1. Stanford Neurosciences Graduate Training Program, Stanford University. Stanford, CA 94305, USA
2. Helen Wills Neuroscience Institute, University of California, Berkeley. Berkeley, CA 94720, USA
3. Department of Psychology, Stanford University, Stanford, CA 94305, USA
4. Department of Psychology, Arizona State University, Tempe, AZ 85287, USA


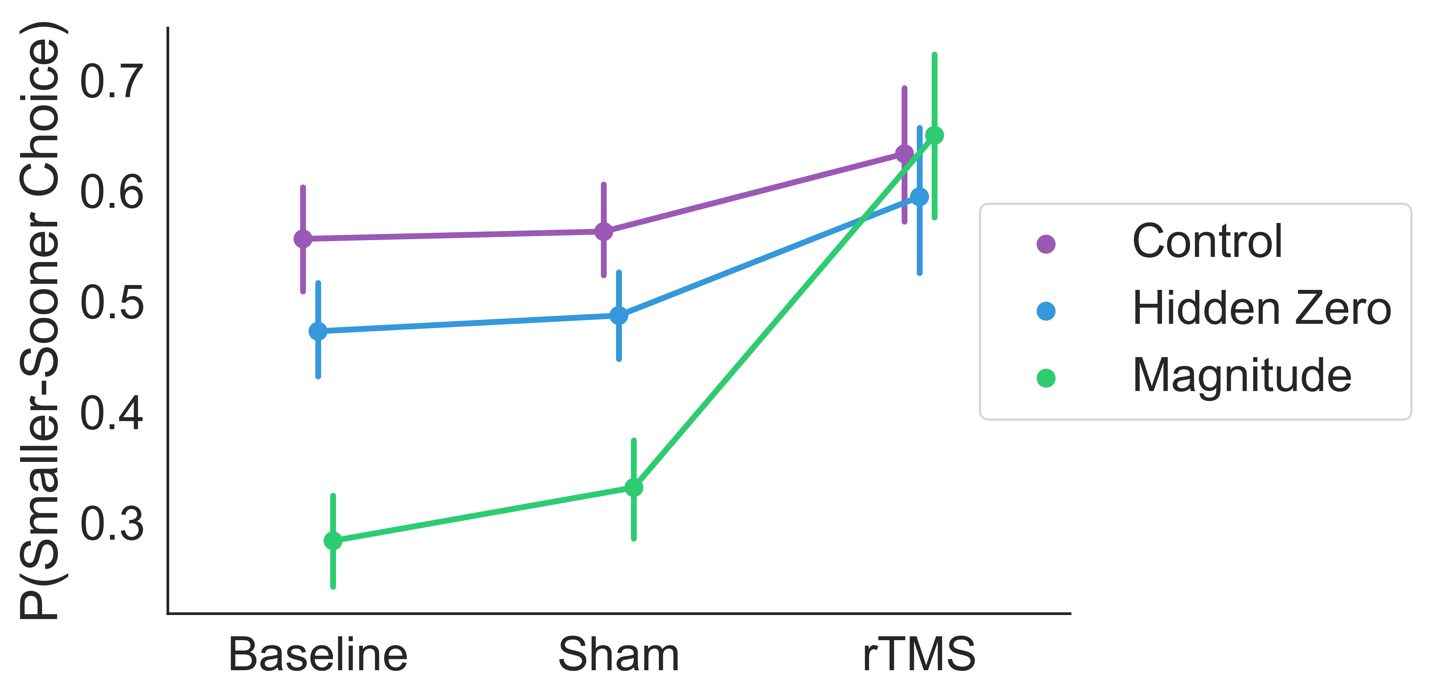


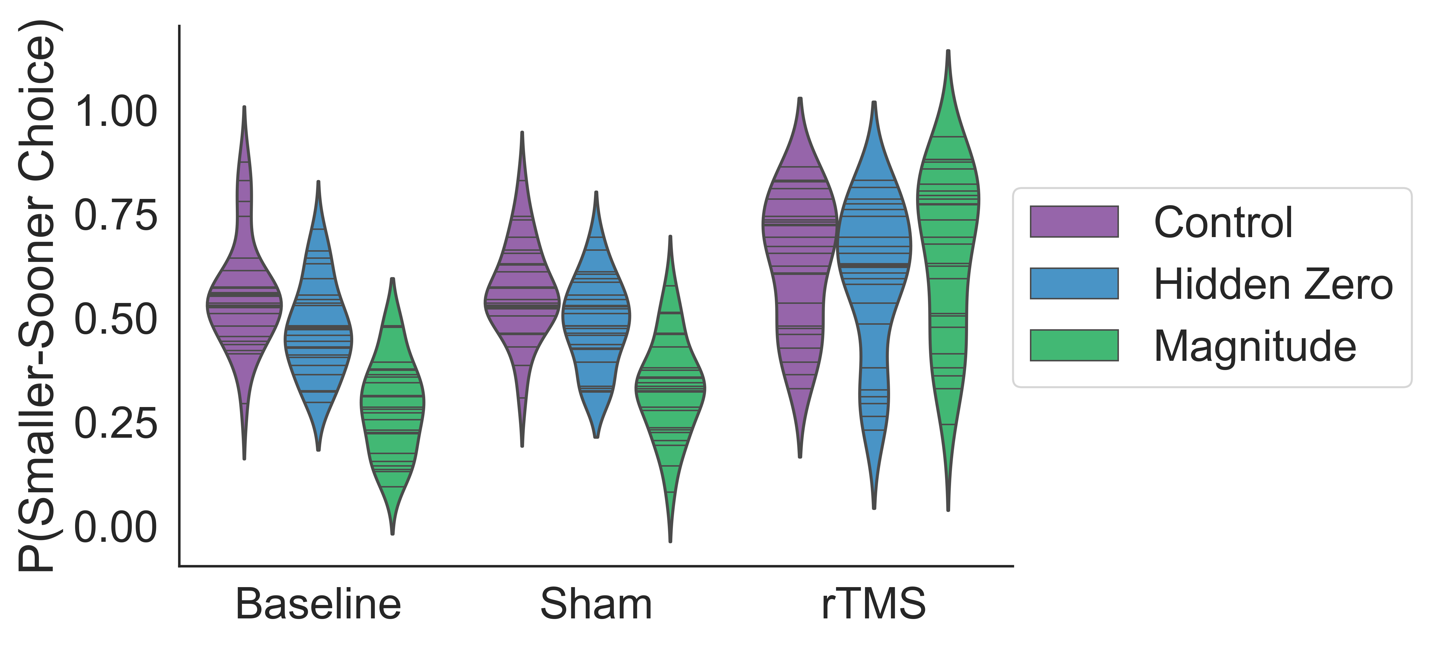


*Figure S1: rTMS over dlPFC eliminates the magnitude effect. Probability of choosing the smaller-sooner option as a function of experimental manipulation. Pre-experiment baseline and sham TMS are the two control sessions. Under baseline and sham, we found that both magnitude and hidden zero framing reduce the number of smaller-sooner choices. rTMS over dlPFC increases the probability of shorter-sooner choices. Further, rTMS increases smaller-sooner choices for high-magnitude more than low-magnitude choices and eliminates the magnitude effect. The lower plot depicts the same relationship while showing individual subject data as bars and distribution information.*

We additionally analyzed our results in a model-free manner by examining the probability of choosing the smaller-sooner option. This approach cannot disentangle the effects of discounting and decision noise; but it has the benefit of being more transparently related to the choice data. We fit mixed-effect ANOVAs exactly as in the main manuscript. Choice probabilities were logit transformed. We found very similar results as the analysis with discount rates in the main manuscript, including all of our magnitude effect results (Table S1). The only qualitative difference between the results is that we found no evidence for an interaction between rTMS and the hidden zero effect for either baseline or sham TMS, *p* > .2 (Table S2). Although this finding is consistent with our original hypothesis, the authors consider the discount rate analysis presented in the main paper to be more sensitive than this analysis. The more sensitive analysis found some evidence for an effect of rTMS on the Hidden Zero effect, and so the authors are not comfortable ruling out that rTMS also reduced the Hidden Zero effect.

Additionally, to more directly replicate results in Figner et al., (2010), we ignored the magnitude and hidden-zero conditions and fit a mixed-effects ANOVA with hemisphere as a between-subject effect and stimulation as a within-subject effect. This analysis revealed a marginal effect of stimulation on discounting with respect to baseline F(1,25) = 4.3, p = .05 , *ƞ_g_*^2^ = 0.07, and a significant effect with respect to sham rTMS, F(1,25) = 5.93, p = .02 , *ƞ_g_*^2^ = 0.03. We did not observe any effects of hemisphere or interactions between hemisphere and stimulation (all p > .2).


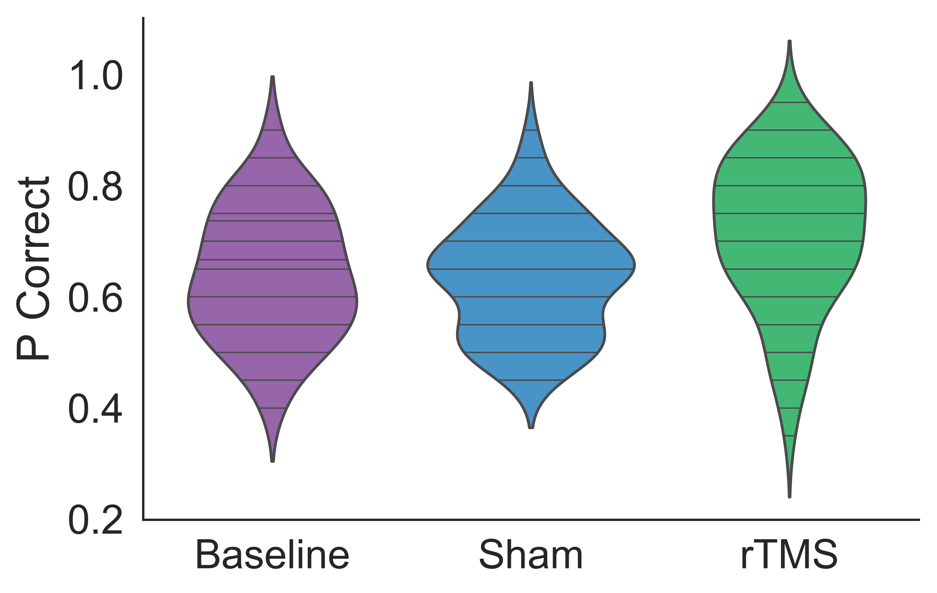


*Figure S2*: *rTMS does not generally disrupt discounting*. We performed a control analysis to Define *k_eq_* to be the value of *k* that would give indifference on a choice. If choices were ordered by *k_eq_*, a subject’s *k* specifies where in this ordered list they switch from preferring smaller-sooner to larger-later rewards. We labeled choices as “correct” if the response was consistent with the discount rate estimated for that subject, and “incorrect” otherwise.


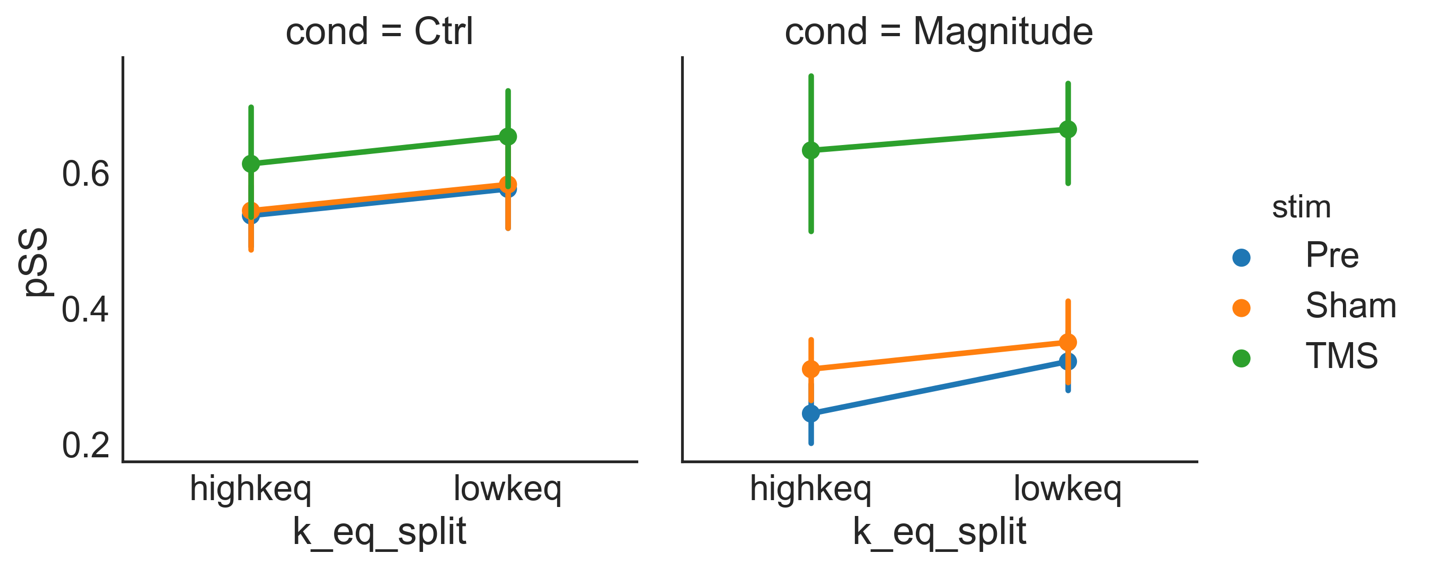


*Figure S3: rTMS biases discounting behavior within-subject without disrupting the relationship between subjective value and choice*. rTMS causes an overall increase in the probability of choosing smaller-sooner without influencing the relationship between *k_eq_* and choice.


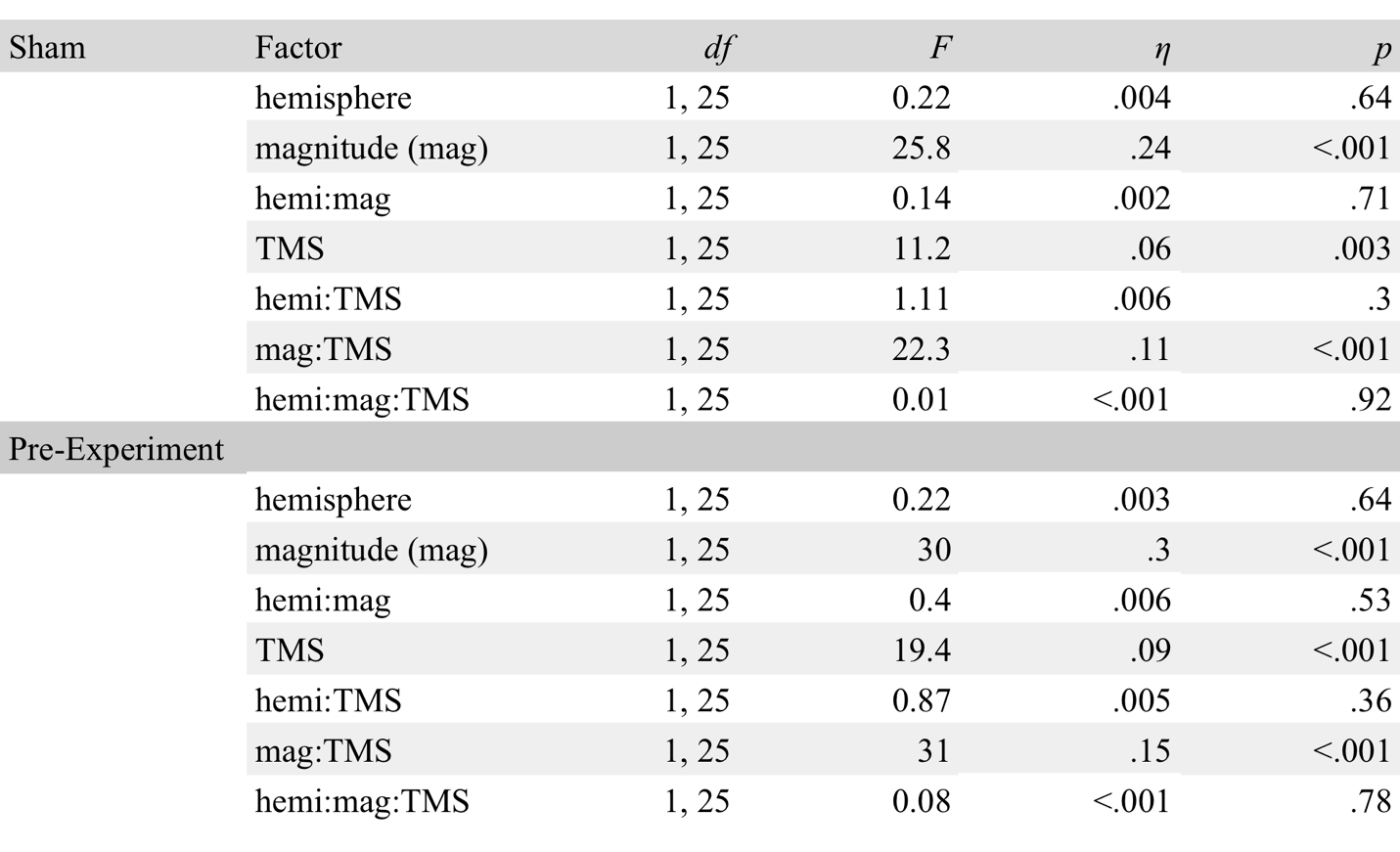


***Table S1.*** *Results of ANOVAs assessing the probability of smaller-sooner choice as a function of magnitude context and rTMS relative to two different control conditions (Sham and Pre-Experiment). Both ANOVAs show significant effects of magnitude, a significant effect of TMS, and a significant interaction between the magnitude effect and TMS. η refers to generalized eta squared.*


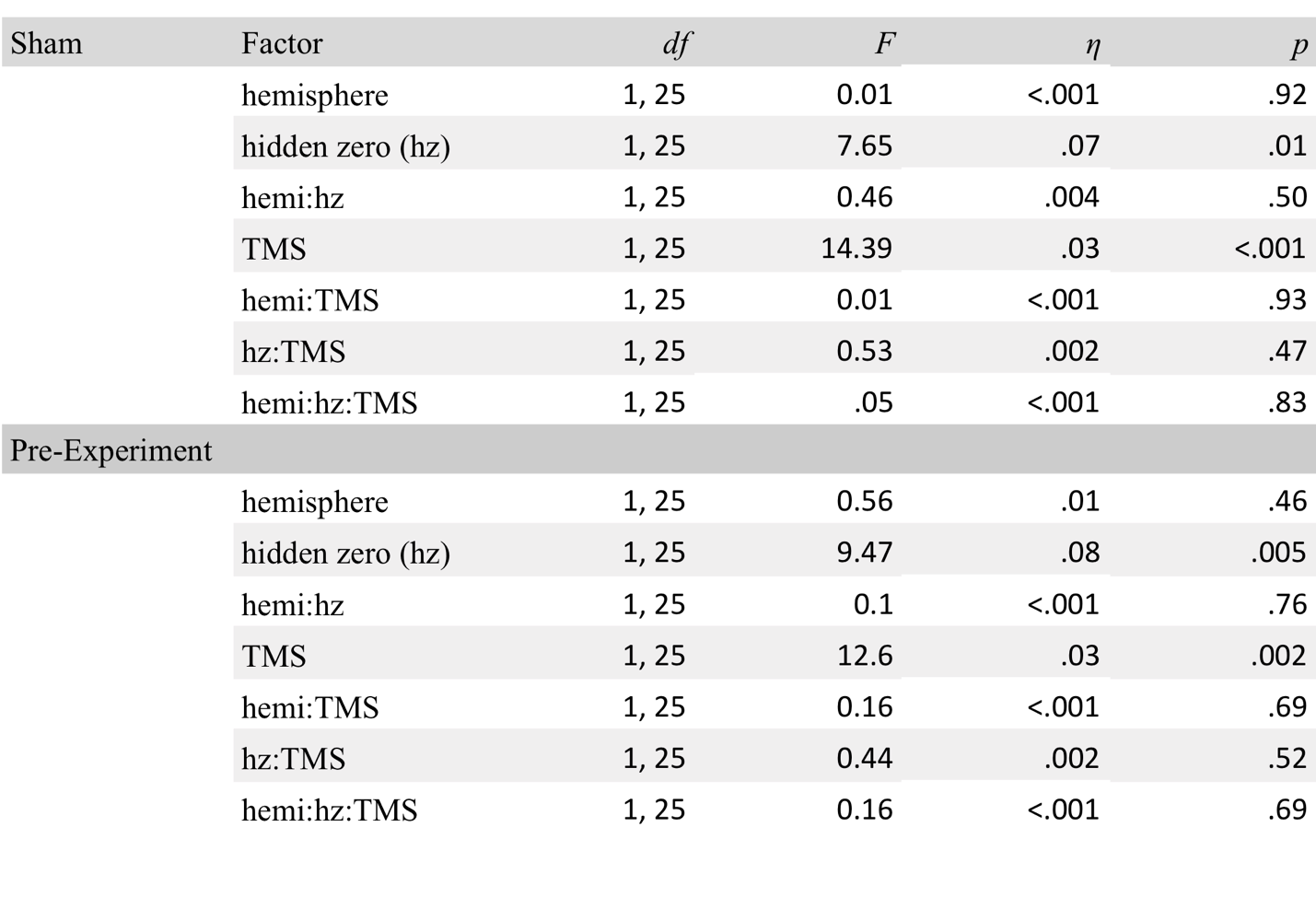


***Table S2.*** *Results of ANOVAs assessing the probability of smaller-sooner choice as a function of hidden zero framing and rTMS relative to two different control conditions (Sham and Pre-Experiment). Both ANOVAs show significant effects of hidden zero framing and rTMS on the probability of smaller-sooner choices. However, neither show evidence of an interaction between rTMS and hidden-zero framing. η refers to generalized eta squared.*
